# Species- and Metal-Specific Responses of the Ionome of Three Duckweed Species under Chromate and Nickel Treatments

**DOI:** 10.3390/plants12010180

**Published:** 2023-01-01

**Authors:** Viktor Oláh, Muhammad Irfan, Zsuzsanna Barnáné Szabó, Zsófi Sajtos, Ágota Zsófia Ragyák, Boglárka Döncző, Marcel A. K. Jansen, Sándor Szabó, Ilona Mészáros

**Affiliations:** 1 Department of Botany, Institute of Biology and Ecology, Faculty of Science and Technology, University of Debrecen, Egyetem Square 1, H-4032 Debrecen, Hungary; 2 Atomic Spectroscopy Partner Laboratory, Department of Inorganic and Analytical Chemistry, Faculty of Science and Technology, University of Debrecen, Egyetem Square 1, H-4032 Debrecen, Hungary; 3 Doctoral School of Chemistry, University of Debrecen, Egyetem Square 1, H-4032 Debrecen, Hungary; 4 Institute for Nuclear Research (ATOMKI), Bem tér 18/c, H-4026 Debrecen, Hungary; 5 School of Biological, Earth and Environmental Science, University College Cork, Distillery Fields, North Mall, T23N73K Cork, Ireland; 6 Department of Biology, University of Nyiregyhaza, H-4401 Nyiregyhaza, Hungary

**Keywords:** duckweed, metal accumulation, ionomics, ICP-OES, micro-XRF

## Abstract

In this study, growth and ionomic responses of three duckweed species were analyzed, namely *Lemna minor*, *Landoltia punctata*, and *Spirodela polyrhiza*, were exposed for short-term periods to hexavalent chromium or nickel under laboratory conditions. It was found that different duckweed species had distinct ionomic patterns that can change considerably due to metal treatments. The results also show that, because of the stress-induced increase in leaf mass-to-area ratio, the studied species showed different order of metal uptake efficiency if plant area was used as unit of reference instead of the traditional dry weight-based approach. Furthermore, this study revealed that μXRF is applicable in mapping elemental distributions in duckweed fronds. By using this method, we found that within-frond and within-colony compartmentation of metallic ions were strongly metal- and in part species-specific. Analysis of duckweed ionomics is a valuable approach in exploring factors that affect bioaccumulation of trace pollutants by these plants. Apart from remediating industrial effluents, this aspect will gain relevance in food and feed safety when duckweed biomass is produced for nutritional purposes.

## 1. Introduction

Duckweeds (Lemnaceae) form a family of small aquatic monocots that usually inhabit the surface zone of slowly moving freshwaters. Their evolutionary adaptation to lentic habitats resulted in specific traits, such as a small, thallus-like body (frond) that displays a much-reduced anatomy [1]. Fronds lack a distinct shoot system and only have adventitious roots. It has long been argued that duckweed roots, and the root-to-shoot transport system, play less of a role in nutrient acquisition compared to most other plant species. Rather, fronds can absorb nutrients through their abaxial (lower) epidermis [2]. Another duckweed feature is the extremely rapid vegetative growth, with 2–3 days-long doubling time under suitable conditions [3]. The high growth rate involves efficient uptake and utilization of nutrients. Duckweeds have been reported to successfully remove different nitrogen forms and phosphorus from various types of waste waters [4,5]. The produced duckweed biomass has a favorable composition with e.g., high protein, starch and fiber content [6]. These traits make duckweeds potent candidate crops for use as biofuel, feed, or food in circular economic approaches [4,7]. In addition, duckweeds are also known to efficiently incorporate many trace elements, and this can potentially make these plants suitable for the remediation of industrial effluents. For example, duckweeds have high accumulation rates for arsenic, boron, cadmium, chromium, copper, manganese, nickel, and zinc [8,9,10,11,12].

When duckweed-based applications are considered, inherent traits of these plants should be taken into account. One of these traits is the high genetic diversity within the family. Thus, although these plants with their reduced morphology may look similar to one another, there is substantial interspecific diversity in traits such as growth potential, biomass composition, metal uptake rates, and/or stress tolerance [5,13,14]. Therefore, selection and matching of the most suitable species for any given application can be crucial. For example, pertaining to metal accumulation, the high affinity of a particular species to a particular trace element can be either advantageous in remediating polluted waters, e.g., [15], or a disadvantage if duckweed is produced for use in feed or food, e.g., [16]. Further, a high metal bioaccumulation rate may not involve high tolerance to the same element that potentially limit the usefulness of certain species in some applications. To even further complicate the situation, besides genotypic differences, mineral acquisition by plants is also influenced by interactions amongst different chemical elements in the medium that may lead to, for instance, uptake competition. Plant ionomics is quantitative and simultaneous analysis of multiple elements in the biomass and is an efficient approach to study the functional state of plants under different conditions [17]. This aspect has, however, been less considered in previous duckweed bioaccumulation studies.

Another consideration for bioremediation applications is that duckweeds are highly plastic plants that will respond to environmental stimuli by rapid phenotypic modulation. In response to changing ambient conditions, duckweeds can acclimate by altering e.g., the frond size or the concentration and ratio of photosynthetic pigments [18,19]. A typical plant response to suboptimal conditions is the increase of dry matter content and leaf (in case of duckweeds frond) mass-to-area ratio (LMA) [20,21,22]. In duckweeds, this response is rapid and may be attributed to several mechanisms, such as disturbed frond development and expansion [23], regulated modulation of frond development [20], or the rapid accumulation of starch in fronds [24]. Any of these mechanisms may trigger a stress-induced increase in frond LMA, and this will be inherently accompanied by slower growth in terms of the expansion of frond area. This can result in seemingly divergent growth rates depending on whether the growth was calculated on dry mass or surface area basis.

In the context of changing frond morphology, a further question is whether the acquired nutrients and/or metals are uniformly distributed within the fronds, or whether internal transport and redistribution lead to metal accumulation in particular frond regions. Previous reports showed that metal uptake was not uniform, and that the node-zone of fronds and roots showed higher uptake rates than other parts of the frond [25,26]. In the case of Cd-treated *Landoltia punctata*, this resulted in Cd accumulation primarily in the node and veins [27]. Thus far, studies have mostly analyzed metal accumulation at subcellular or tissue levels in duckweed fronds and roots [28,29]. Frond-level distribution is considered less often. Recently, various methods have become available to map elemental distributions in biological samples (e.g., histochemical assays, energy dispersive X-ray fluorescence microprobe—i.e., μXRF, and laser ablation plasma mass spectrometry—i.e., LA–ICP–MS). The thin, two-dimensional structure of fronds makes duckweeds particularly suitable for such analyses.

The aim of this study was to analyze the ionome of duckweed biomass in three species of Lemnaceae under either optimal growth conditions or following chromate or nickel treatments. More specifically, we tested (i) the species- and treatment-specific patterns in the ionome, (ii) whether the apparent order of species in metal accumulation efficiency was affected by the unit of reference (i.e., dry mass v. area), and (iii) if the within-frond distribution of the acquired Cr and Ni showed distinct patterns of accumulation or was rather uniform.

## 2. Materials and Methods

### 2.1. Culturing Conditions and Experimental Setup

Axenic stocks of the three studied duckweed species, *Spirodela polyrhiza* L. Schleid. (clone UD0401), *Landoltia punctata* (G. Meyer) Les & Crawford (Landolt clone #7760), and *Lemna minor* L. (clone UD0201) were maintained in 300 mL Erlenmeyer flasks on modified Steinberg medium (pH 6.0 ± 0.2, [30]) under constant temperature (24 ± 2 °C) and irradiation (PPFD 60 ± 10 μE m^−2^ s^−1^, white) [31].

For the experimental work, healthy colonies of 7–8 days old stock cultures were used. The metal treatments were conducted in crystalizing dishes (80 mm diameter and 150 mL volume) and covered with plastic Petri dishes to reduce evaporative loss. Each vessel contained 100 mL of Steinberg medium either without supplemental metal (control), or with nominal added concentrations of 4 mg L^−1^ Cr(VI) (K_2_Cr_2_O_7_ salt) or 2.5 mg L^−1^ Ni (NiSO_4_ · 7 H_2_O salt), respectively. The applied metal concentrations were based on our previous work with the same *S. polyrhiza* clone [31] and aimed at resulting in significant growth inhibition without leading to frond mortality by the end of the experiments. For preparing the Steinberg medium and metal treatments, reagent grade chemicals and Type I ultrapure water were used.

The starting inoculum was 2.7 ± 0.6 cm^2^ frond area per vessel for *S. polyrhiza*; 1.5 ± 0.4 cm^2^ for *La. punctata* and 2.3 ± 0.7 cm^2^ for *Le. minor*, corresponding to an initial biomass of 62.0 ± 21.9 mg FW (6.5 ± 1.3 mg DW) for *S. polyrhiza*; 58.1 ± 16.5 mg FW (5.0 ± 1.2 mg DW) for *La. punctata* and 71.1 ± 7.8 mg FW (6.3 ± 1.5 mg DW) for *Le. minor*.

The metal treatments lasted for 72 ± 2 h under ambient conditions identical to those used for stock culturing. Every treatment for each species was performed in triplicate and was repeated in two independent experiments.

### 2.2. Measurement of Growth

On the starting (0th) and final (3rd) day of experiments, digital images of each vessel were recorded by means of a custom-made photo hood and a PC-controlled camera (5 MP resolution) mounted in a perpendicular position to the surface of the cultures. Frond area (FA) in individual vessels was then determined from the obtained images by means of “Threshold colour” and “Analyze particles” functions of ImageJ image analysis software [32].

On the final day of experiments, the plants were harvested, thoroughly rinsed with Type I ultrapure water, and gently blotted dry with paper towels. The fresh (FW) and dry weight of plants (DW) in each vessel was determined using an analytical balance (Kern ABT 120-5DM). Prior to determination of DW, the plants were dried until constant weight (4 days at 65 °C).

We calculated the initial biomass using the frond areas in each vessel on the 0th day, and the average species-specific leaf mass-to-area ratios (LMA as mg DW cm^−2^) and dry matter contents (DM%) of plants in the same stock cultures that were used for the experiments (*n* = 8 per species).

Based on the obtained biomass data, the following parameters were calculated:

Dry matter content: DM% (%) = (DW/FW) ∗ 100

Relative growth rate: RGR_x_ (day^−1^) = (lnX_j_ − lnX_i_)/t

where X_i_ and X_j_ denote the respective parameter (FA, FW, DW) determined on the ith (0th) and jth (3rd) days of experiments, and t is the treatment duration (3) in days, according to [33].

Growth inhibition: I% = ((RGR_control_ − RGR_treated_)/RGR_control_) ∗ 100

where RGR_treated_ is the relative growth rate of a metal-treated culture and RGR_control_ is the corresponding mean control relative growth rate, according to [33].

### 2.3. Determination of Metal Content in the Growth Medium and Biomass

For the analytical determination of metallic elements in the medium, 10 mL of medium from each vessel was filtered through a 100 µm pore size nylon mesh filter (SPL Life Sciences) on the 0th and 3rd days of the tests. On the 0th day, the samples were taken from the solutions prepared for control, Cr(VI) and Ni treatments in triplicates, while on the 3rd day from the media of each experimental vessel. The samples were immediately acidified with 2 drops of 65% (m/m) HNO_3_ (reagent grade, Scharlau), and stored at room temperature prior to elemental analysis.

In analysis of biomass composition, 3–10 mg of the dried plant samples (see Section 2.2) was digested under atmospheric pressure in a mixture of 3.0 mL 65% (m/m) HNO_3_ (reagent grade, Scharlau) and 1.0 mL 30% (m/m) H_2_O_2_ (reagent grade, Merck). Then, the digested extracts were transferred without loss into volume-calibrated plastic centrifuge tubes and diluted to a final volume of 5 mL with ultrapure water (Synergy UV Millipore). The solutions were stored at room temperature prior to further elemental analysis.

Metal concentration of medium and biomass samples were determined by means of inductively coupled plasma optical emission spectrometry (ICP-OES 5110 Vertical Dual View, Agilent Technologies, Santa Clara, CA, USA). An auto-sampler (Agilent SPS4), Meinhard^®^ type nebulizer and double-pass spray chamber were used. The ICP-OES operating conditions and measurement parameters to determine the elemental concentrations of Al, B, Ba, Bi, Ca, Cd, Co, Cr, Cu, Fe, K, Li, Mg, Mn, Na, Ni, Pb, Sr, and Zn are provided in Tables S1 and S2. Standard solutions of the macro elements (Ca, K, Mg, and Na) were prepared from a mono-element spectroscopic standard of 1000 mg L^−1^ (Scharlau), while those of the micro elements (Al, B, Ba, Bi, Cd, Co, Cu, Cr, Fe, Li, Mn, Na, Ni, Pb, Sr, and Zn) were similarly prepared from the multi-element spectroscopic standard solution of 1000 mg L^−1^ (ICP IV, Merck). In both cases, a five-point calibration process was used, in which the standard solutions were diluted with 0.1 M HNO_3_ prepared in ultrapure water.

Due to their low concentrations in both sample types (i.e., medium and biomass), Al, Ba, Bi, Cd, Co, Li, and Pb were excluded from the subsequent data processing.

The measured metal concentrations in the medium and biomass were used to calculate the metal- and species-specific bioconcentration factors (BCF) for the analyzed elements as follows:

BCF = Cc_biomass_/Cc_medium_

where Cc_biomass_ and Cc_medium_ were the measured concentrations of a given element in the duckweed biomass (mg kg^−1^ DW) and in the medium (mg L^−1^), respectively.

The measured Cr(VI) and Ni contents in the biomass were also transformed into metal content per unit frond area, as follows:

metal content (mg m^−2^) = (Cc_biomass_ ∗ DW)/(FA ∗ 100)

where Cc_biomass_ was the measured concentration of a given element in the duckweed biomass (mg kg^−1^ DW), DW was the harvested dry weight (mg), and FA is the frond area corresponding to the harvested biomass (cm^2^)

### 2.4. mXRF Analyses

For the elemental mapping of metal distribution within fronds, the sample plants were subjected to the same Cr(VI) and Ni treatments and placed under the same ambient conditions as described in Section 2.1. Prior to μXRF scanning, the plants were transferred from metal-containing medium to 50 mL Type I ultrapure water for 10 min in order to remove excess metals adhering to the external surface of fronds and roots. Following that, the roots were carefully removed, and the plants were air-dried for 3 days, while being gently pressed to prevent deformation of fronds.

µXRF investigations were carried out using a Bruker M4 TORNADO Micro-XRF spectrometer (Bruker, Billerica, MA, USA) using a Rh-tube without any filter, at 50 kV accelerating voltage and 400 µA current. Characteristic X-ray lines were recorded by two energy dispersive detectors. Each of the two Be-window silicon drift detectors had a 30 mm^2^ active area. The mapping was performed in 20 mbar vacuum. The beam diameter was focused to 20 µm by the built-in polycapillary lens. The recorded rectangular maps were acquired with 100 ms/pixel velocity and two accumulations. For QMap analysis, the M4 TORNADO software (version: 1.6.621.0) was used.

### 2.5. Data Processing and Statistics

To analyze species-specific and metal-induced growth and ionomic responses, respective data of the two independent experiments per species and per treatment were pooled, resulting in a sample size of *n* = 6 for each combination. Multiple comparisons were performed by means of Kruskal–Wallis test and, in case of significant differences, with *post hoc* Mann–Whitney pairwise comparisons of medians. The biomass ionomic dataset was also subjected to multivariate analysis by means of principal component analysis (PCA). All statistical analyses were performed by means of Past v4.0 [34], and for every analysis, <5% probability, that is, *p* < 0.05, was considered as statistically significant.

## 3. Results and Discussion

### 3.1. Metal-Induced Growth Inhibition

**Figure 1:**
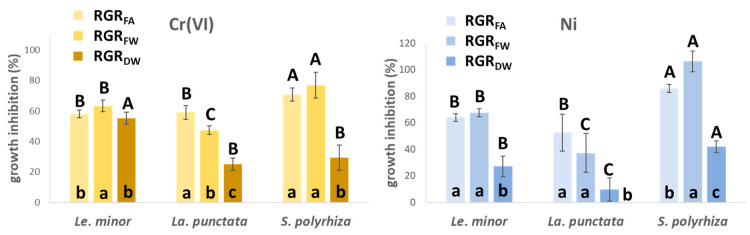
Cr(VI)- (**left**), and Ni-induced inhibition (**right**) of relative growth rates (RGR) derived from different growth parameters (frond area—FA, fresh weight—FW, dry weight—DW). The inhibition was compared to the respective species-specific control growth rates. Means ± SD of *n* = 6 samples; different lower cases at the bottom of bars denote significantly different (*p* < 0.05) inhibition of growth parameters with respect to the given species (*Le. minor*, *La. punctata* or *S. polyrhiza*) and treatment, while different capitals on top of the bars indicate significantly different (*p* < 0.05) growth inhibition of species based on the same growth parameter.

The observed trends indicate that, in general, the applied modified Steinberg medium can support normal growth of duckweeds. As expected, controls displayed good growth with RGR values ranging between 0.3 and 0.4 day^−1^ for every species and growth parameter. In general, the three species showed similar growth responses to Cr(VI) treatments, with a slightly larger frond area- and fresh weight-based growth inhibition for *S. polyrhiza* (I% = 70–77%) and a stronger dry weight-based growth inhibition (I% = 55%) for *Le. minor* (Figure 1). To Ni treatments, on the other hand, *S. polyrhiza* showed considerably greater sensitivity with almost arrested frond area (I% = 86%) and fresh weight growth (I% = 106%), while *La. punctata* proved to be slightly more tolerant (I% = 10–53% for the three growth parameters) than *Le. minor* (I% = 27–63% for the three growth parameters, Figure 1). It has previously been reported that different duckweed species may have different sensitivity to metals [35,36], and the same order of Ni-sensitivity amongst the three species was reported by Xyländer and Augsten [37]. Similarly, Appenroth et al. [38] found *S. polyrhiza* to be more Ni-sensitive than *Le. minor*. Thus, experimental data support our finding that *S. polyrhiza* is highly sensitive to Ni.

In general, RGR_FA_- and RGR_FW_-based growth inhibitions showed high similarity to each other (Figure 1). In contrast, I% based on RGR_DW_ indicated considerably lower growth decline in most cases than the former parameters. The reason for the diverging responses of RGR_DW_ versus RGR_FA_ and RGR_FW_ is in part the altered phenotype of newly formed fronds. Metal-induced disorders and morphogenic responses in frond expansion are known to increase LMA and DM% in duckweeds [23,38]. In *Le. minor*, DM% increased by 24 (Cr(VI) and 76% (Ni), in *La. punctata*, by 25 (Cr(VI) and 38% (Ni), and in *S. polyrhiza*, by 41 (Cr(VI) and 58% (Ni) in just 3 days (Table 1). This increase in DM%, to some extent, diminished the metal-induced decline in growth of dry matter of cultures. Apart from morphogenic modifications, elevated DM% can also be attributed to the accumulation of starch [24]. Rearranged carbon utilization to produce starch instead of new frond area is common amongst duckweeds when exposed to trace metals [13,39]. Ni proved to be particularly efficient in enhancing starch accumulation in duckweeds [40], and chromate is also known to have a similar effect [23].

### 3.2. Changes in the Composition of the Nutrient Medium

**Figure 2:**
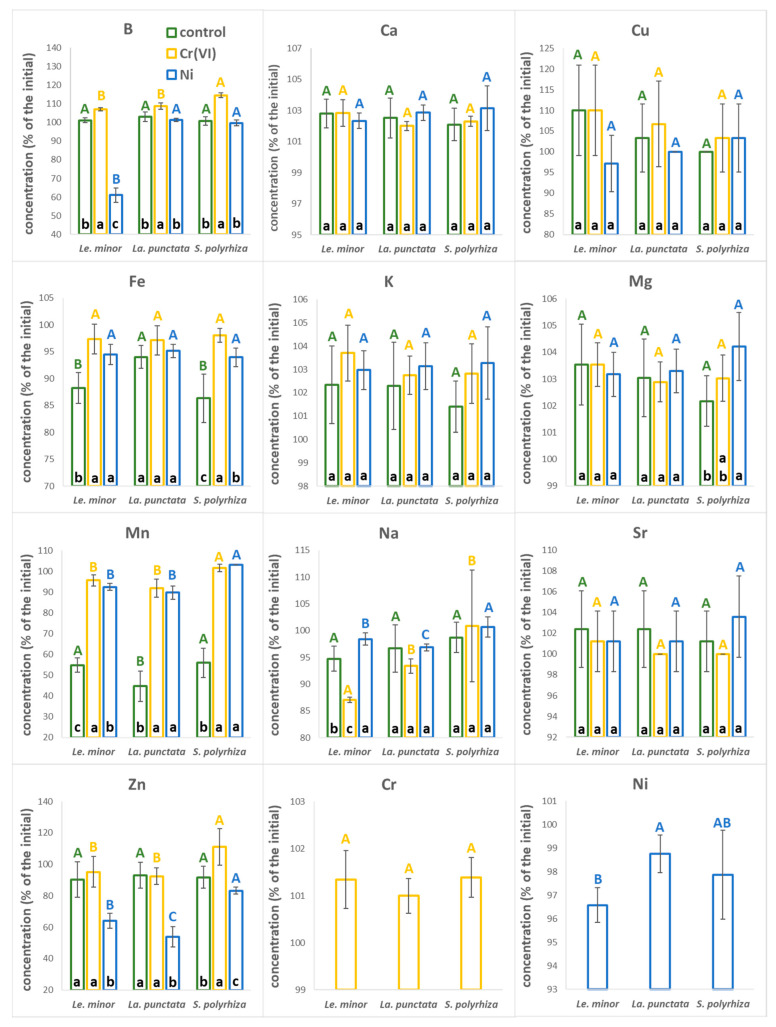
Ionic composition of the applied nutrient medium at the end (3rd day) of the experiments in control (green), Cr(VI) (yellow) and Ni treatments (blue). The data denote percentile concentrations as compared to the initial medium composition on the 0th day of the treatments. Means ± SD of *n* = 6 samples are shown. Different capitals with corresponding colors indicate significantly different (*p* < 0.05) medians of species across the same treatment. Different lower cases in the bottom of bars indicate significantly different (*p* < 0.05) medians of different treatments in case of the same species. The measured ionic concentrations are provided in Table S3.

In general, the concentrations of most analyzed elements in the medium did not change much under control (no metal supplementation) conditions over the test duration (Figure 2, the actual concentrations are supplemented in Table S3). However, a substantial drop was found in the concentration of Mn, which had decreased by 44% (*S. polyrhiza*), 45% (*Le. minor*), and 55% (*La. punctata*) by the end of tests (Figure 2). In Cr(VI) treatments, the medium composition changed even less compared to that of the control (Figure 2). In fact, the concentration of most elements (B, Ca, Cu, K, Mg, and Sr) slightly increased, probably due to the evaporative loss. Similarly, chromium level stayed similar (101%) to its original concentration (Table S3). In Ni treatments, the concentration of B in the medium decreased strongly in the case of *Le minor* (−39%), while it stayed unchanged (99–101%) in case of the other two species (Figure 2). The other element that showed considerable depletion was Zn with a 27% (*S. polyrhiza*) to 46% (*La. punctata*) decrease in concentration compared to the initial content. The concentration of the supplied Ni decreased only marginally (by 2–3%) by the end of treatments (Table S3).

Duckweeds have been reported as efficient accumulators of Mn [41]; and the fact that ~50% of this element was taken up by the control plants in just 3 days, points to the possible depletion from the medium during extended longer-term experiments under static conditions. Yet, Mn deficiencies are somewhat unlikely as duckweed colonies seem to have the ability to transport and redistribute metallic elements among and within fronds (see later in Section 3.5). Such an internal translocation may counterbalance the decreasing Mn supply from a depleting medium, even in Steinberg medium that represents the lower end in terms of Mn-content amongst the most frequently applied duckweed media [30]. Interestingly, both Cr(VI) and Ni treatments disrupted the enhanced uptake of Mn from the medium. Ni is known to affect Mn acquisition of both crops and hyperaccumulator plants by competing for uptake [42,43]. Cr(VI), however, forms oxyanions, and thus enters plants via different routes [44]. Previous literature is not consistent and report both synergistic and antagonistic effects of chromate treatments on Mn content of plants [45,46]. Park [44] suggested that Cr(III) can reduce Mn in the soil thus increasing its bioavailability. Reversing this logic, it can be speculated that Cr(VI) in turn might oxidize Mn, limiting its uptake by plants. Facilitated removal of B and Zn by *Le. minor* and *La. punctata* under Ni treatments indicates that wider nutrient uptake patterns of plants can be affected following metal treatment. A synergistic increase in the uptake of Zn in the presence of Ni was previously explained by up-regulated Zn-transporters in response to a virtual Zn-deficiency due to the substitution of this metal by Ni at functional binding sites, or by a disrupted sensing machinery for Zn uptake [47].

### 3.3. Biomass Ionomic Composition

The plant ionome is regulated by both taxonomic and environmental factors, and multi-elemental analyses can provide important additional information on the nutrient uptake preferences or functional state of the studied organisms [17]. Previously it has been demonstrated, as an example, that co-occurring, closely related Ericaceae species had distinct ionomic profiles that may be considered as a strategy to alleviate competition in densely populated habitats with high taxonomic diversity [48]. Considering duckweed mats as analogously “crowded” habitats, diverging nutrient preferences of species—or intraspecific genotypes—may thus be hypothesized supporting higher productivity of both natural and man-made (e.g., in constructed wetlands) duckweed polycultures.

**Figure 3:**
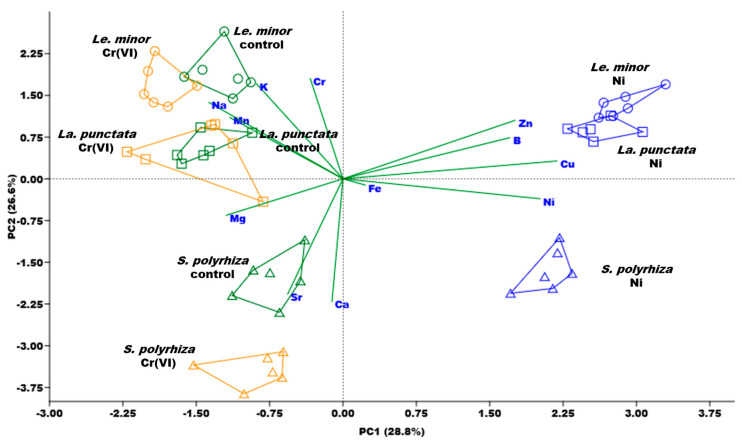
Overall patterns in the biomass ionomic composition of the studied duckweed species according to the first two principal components (PC1 and PC2, respectively) of the performed principal component analysis. Polygons delineate *n* = 6 samples of different treatments (control—green; 4 mg L^−1^ Cr(VI)—orange; or 2.5 mg L^−1^ Ni—blue) of the three species (*Le. minor*—circles, *La. punctata*—squares, *S. polyrhiza*—triangles).

According to the multi-elemental analysis, the first two principal components covered 55.4% of the overall variance in the ionomic dataset (Figure 3, the actual concentrations in the biomass and the comparative statistics are supplemented in Table S4). PC1 correlated strongly with Ni contents. Besides Ni, high positive correlations of this principal component were also found for B, Cu, and Zn (Table S5). Mg, Mn, and Na, in contrast, showed a weak negative correlation with PC1. Cr content of the biomass correlated positively with PC2, similarly to K (Figure 3). Ca and Sr, on the other hand, had negative correlations with that principal component (Table S5). The three studied duckweed species were also mostly separated along PC2 (Figure 3). This distribution suggests that genotype-specific differences in the biomass composition can be hypothesized even in these closely related species. From that aspect, *Le. minor* can be characterized as having higher K and Na contents than *La. punctata* and especially *S. polyrhiza* (Table S5). The latter species, on the other hand, had higher Ca and Sr contents. Being chemical analogues, the more efficient Ca uptake by *S. polyrhiza* compared to *La. punctata* and *Le. minor* can explain the higher Sr content in the biomass of this species as well [49].

**Figure 4:**
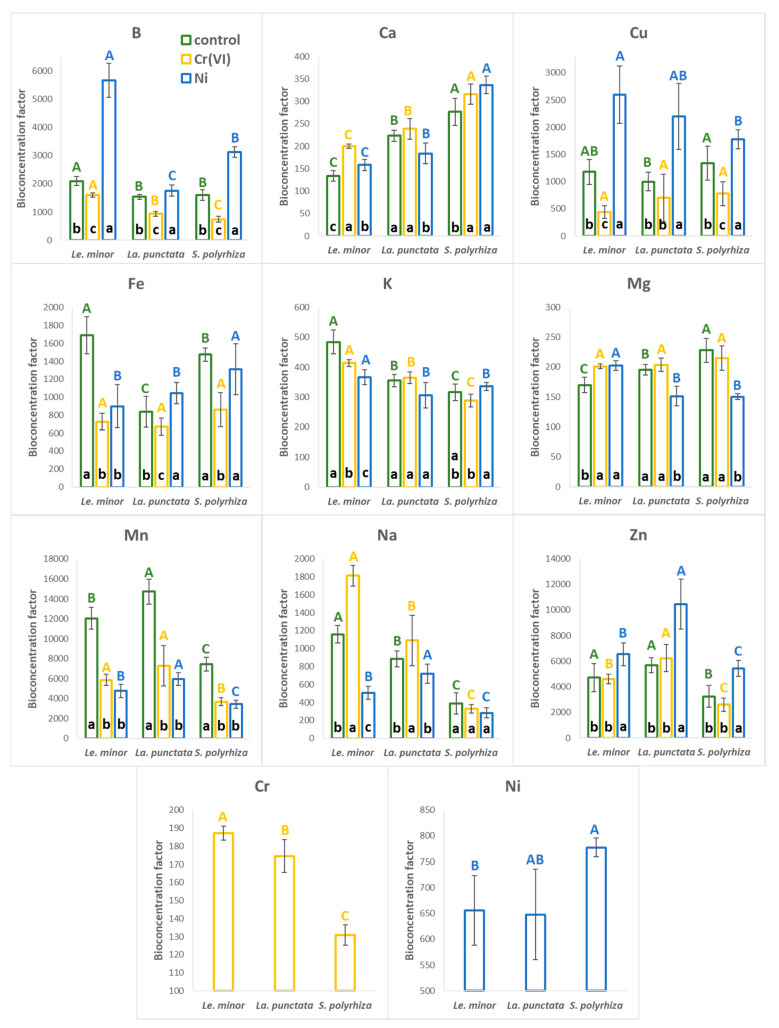
The calculated bioconcentration factors of the analyzed elements under control (green), Cr(VI) (orange), and Ni treatments (blue), in the three duckweed species. Means ± SD of *n* = 6 samples are shown. Different capitals with corresponding colors indicate significantly different (*p* < 0.05) medians of species across the same treatment. Different lower cases in the bottom of bars indicate significantly different (*p* < 0.05) medians of different treatments in case of the same species. Note: Results for Sr are not presented because the concentration of this element was below the detection limit in several samples. The original elemental concentrations have been supplemented in Table S4.

The calculated bioconcentration factors (BCF) under control conditions indicate that the three tested duckweed species accumulated moderate concentrations of K, Na, Ca, and Mg, with BCF values in the range of ~130–1200 (Figure 4). The relatively high BCFs of the non-essential element Na can be explained by its low ambient concentration in the medium. However, the studied species seemed to accumulate Na differently (Table S4): *Le. minor* showed the highest BCF value (1159 ± 97), while *La. punctata* showed 24% lower (885 ± 89), and *S. polyrhiza* 67% showed lower (396 ± 118) bioaccumulation factors. Compared to Na, Ca, Mg, and K were more abundant in the medium and showed lower BCF values (~130–280 for Ca, ~170–230 for Mg and ~315–485 for K). As a result, those four elements comprised an average of 6.9 (*S. polyrhiza*), 7.2 (*La. punctata*), and 8.9% (*Le. minor*) of the dry biomass in the three species under control conditions (Table S4).

B, Cu, Fe, and Zn typically showed one order of magnitude higher range of BCF (~800–5700) compared to K, Na, Ca, and Mg, while the highest BCF in all the tested species was calculated for Mn (BCF = ~7500–15000, Figure 4). Duckweeds have long been known to efficiently accumulate various trace elements [2,50], and particularly high bioaccumulation capability was reported, amongst others, for B, Cu, Zn, and Mn [8,11,15,41,51,52,53]. Our data are in line with those observations and indicate that in the applied Steinberg medium, Mn was by far the most efficiently incorporated essential metal (Figure 4 and Table S4), explaining its rapid decline in the medium.

In the Cr(VI) treatment, Cr was accumulated with a relatively low efficiency; its BCF ranged from ~130 (*S. polyrhiza*) to ~190 (*Le. minor*). Similarly, in the Ni treatment, Ni had a fairly low BCF (~650–780), lower compared to e.g., Cu and Fe (Figure 4). Relatively low bioaccumulation factors can be explained by the high abundance of Cr(VI) and Ni in the medium. BCF in general tends to decrease with increasing ambient concentrations of the respective element due to homeostatic or protective regulation of uptake and cellular concentration [54,55]. The calculated BCFs fit well in the range reported previously for duckweeds under comparable ambient concentrations. Literature data delineate the typical BCF range in duckweeds as ~50–300 for Cr(VI) [9,56,57], and ~100–670 for Ni [10,52,58]. Thus, one can hypothesize that these BCFs are characteristic to duckweeds in general. Comparing the two applied metals, our results are in line with literature data and suggest higher affinity of duckweeds for Ni than for Cr(VI), and this can be partly explained by the essentiality of the former element.

As the PCA indicated, the exposure to and uptake of Cr(VI) and Ni altered the mineral composition of the biomass in terms of other elements. In Ni treatments, BCF of B, Cu, and Zn increased by 30–170% compared to the control, while that of Mn was reduced by 54–60% in all three species (Figure 4). In Cr(VI) treatments, on the other hand, BCF of B, Cu, Fe, and Mn showed a generally decreasing trend with a 20–72% drop. Meanwhile, BCF of Ca slightly increased (+7–50%) due to Cr(VI) treatment as compared to control. Changes in the bioaccumulation of Na were species-specific, rather than metal-specific (Figure 4).

It should be noted that even smaller changes in the biomass ionic concentrations of elements strongly affected the relative ratios of elements in the biomass of different species. For example, Cr(VI) treatment increased the Ca/Mg ratio from 4.2 ± 0.1 to 5.4 ± 0.2 (+27%) in *Le. minor* and from 6.5 ± 0.3 to 8.0 ± 0.5 (+22%) in *S. polyrhiza* compared to control, while in *La. punctata* it stayed similar (6.3 ± 0.5, i.e., +3%) to control (6.2 ± 0.1). The Ca/Mg ratio increased by 84% (to 12.0 ± 0.5) in *S. polyrhiza* due to Ni treatment, while it did not change significantly in the other two species (4.2 ± 0.3 and 6.5 ± 0.2 in *Le. minor* and *La. punctata*). In turn, this may have consequences for growth, as the Ca/Mg ratio is an important determinant of plant health [59]. K/Na ratios showed opposite trends in case of Cr(VI) and Ni, mostly due to the changes in Na-uptake (Figure 4). Under control conditions, this ratio was 107.3 ± 7.3 in *Le. minor*, 103.6 ± 9.2 in *La punctata*, and 226.9 ± 69.2 in *S. polyrhiza*. Treatments with Cr(VI) decreased the K/Na ratio to 36.4 ± 3.1 (−66%), to 54.8 ± 7.5 (−47%) and to 141.9 ± 19.5 (−37%) in *Le. minor*, *La. punctata* and *S. polyrhiza*, respectively. Ni, on the other hand, increased this ratio to 185.6 ± 16.4 (+73%) and to 311.5 ± 60.9 (+37%) in *Le. minor* and *S. polyrhiza*, while *La. punctata* was not affected significantly (109.4 ± 15.3, +5.5%, *p* > 0.05). Similarly to the Ca/Mg ratio, K/Na balance in plant tissues may play an important role, especially under saline conditions. Being the most abundant cation in plant cells, controlled uptake and accumulation of K^+^ are crucial in many physiological processes, including osmoregulation, enzyme activities and membrane polarization [60].

### 3.4. Cr and Ni Accumulation

**Figure 5:**
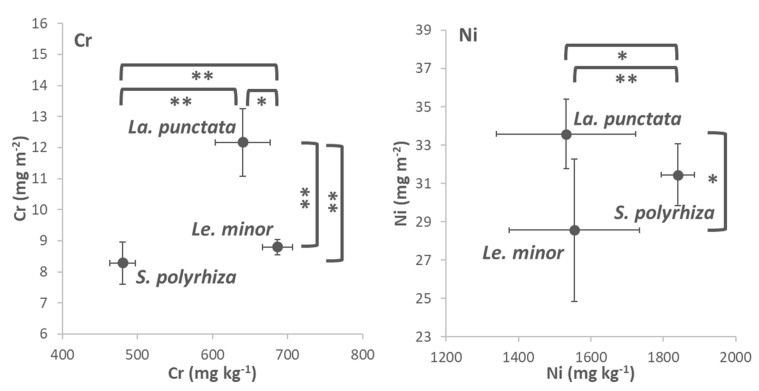
Cr (**left**) and Ni (**right**) contents in the biomass of the three tested duckweed species, expressed on dry weight- (*x*-axes) and area basis (*y*-axes). Means ± SD of *n* = 6 samples are shown. Asterisks denote significant differences between the respective species at * *p* < 0.05 and ** *p* < 0.01 probability levels according to the performed Kruskal–Wallis test and *post hoc* Mann–Whitney comparisons.

The dry mass-based metal contents indicated that most Cr was accumulated by *Le. minor* (Figure 5 and Table S4). Compared to this species, *La. punctata* and *S. polyrhiza* accumulated 7 and 30% less Cr after 3 days of exposure. The most Ni on a dry weight basis was accumulated by *S. polyrhiza*, while the two other duckweed species contained 16–17% less of this metal by the end of the Ni-treatments (Figure 5 and Table S4). The frond area-based metal contents showed a different order of the species. On this basis, *La. punctata* proved to have the highest Cr-and Ni-contents at the end of the metal treatments, though in the latter case it was not significantly higher than that of *S. polyrhiza* (Figure 5). *Le. minor* accumulated 28% less Cr and 15% less Ni on area basis, while *S. polyrhiza* accumulated 32 and 6% less Cr and Ni after 3 days of exposure, respectively. These results highlight that the apparent interspecific differences in metal accumulation can not only be attributed to the mere efficiency of uptake mechanisms (e.g., membrane transport, compartmentation), but also depend strongly on the biomass basis used for quantification. This can be explained partly by inherent differences in the species-specific LMA. In addition, the observed interspecific differences suggest the influence of species-specific changes in DM% and LMA due to metal stress. This way, stress-induced morphogenic responses and increases in starch content can bias the apparent metal uptake efficiency. Therefore, it is advisable to extend the scope of ecotoxicological studies to the area-based metal accumulation capability, and this is particularly important when duckweeds are considered as bioremediating agents [12,53]. Such an approach is widely used when nutrient removal by duckweeds is studied [4,61], but typically lacks in studies that focus on potential reclamation of metal-contaminated waters.

### 3.5. Within-Frond Distribution of Cr and Ni

**Figure 6:**
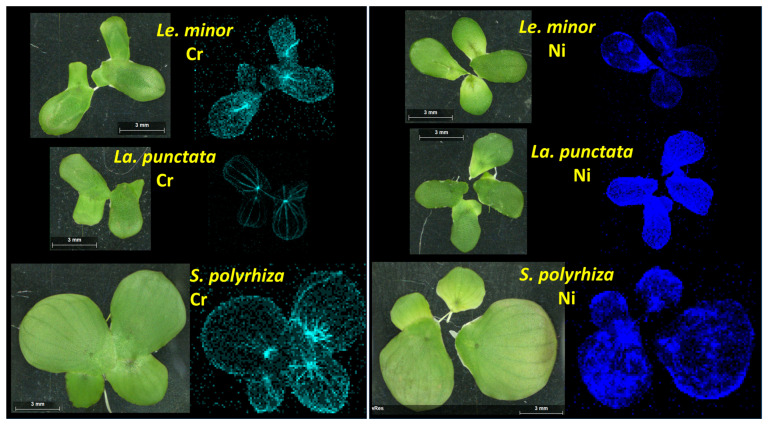
The distribution of Cr (**left**) and Ni (**right**) within the fronds of different duckweed species according to the μXRF maps. The color intensity is proportional to the elemental concentration of Cr (light blue) and Ni (blue). Note: the contrast in elemental maps was digitally enhanced after scanning.

The obtained elemental maps show that μXRF is suitable for the study of internal translocation and distribution of various metals in duckweeds (Figure 6). Considering the popularity of these plants in impact studies and water reclamation, this method may offer a better understanding of the interactions between the duckweed frond and its environment. The μXRF elemental maps revealed that the two metals studied distributed differently within the fronds. Cr primarily accumulated in the veins; the nodes contained particularly large amounts of this metal in all three species (Figure 6). Besides that, frond margins were areas of enhanced Cr accumulation too, especially in *Le. minor* and *S. polyrhiza*. In addition, it should be noted that in these two species, the mesophyll also contained detectable amounts of Cr. This was especially the case in *Le. minor*. In *La. punctata*, on the other hand, Cr accumulation was almost exclusively limited to the vascular tissues and the frond edges (Figure 6). In contrast with Cr, Ni distributed rather evenly in the mesophyll of fronds, with slightly higher concentrations in the frond margins. In *S. polyrhiza*, the node also contained a higher concentration of Ni. In *Le. minor* and *La. punctata*, on the other hand, basal regions of fronds, especially those of daughter fronds, contained elevated Ni contents (Figure 6).

Analogous patterns in terrestrial plants, that is, interveinal chlorosis, damaged leaf margins and tips [62,63], support our finding that vascular tissues play major role in internal translocation of Cr(VI) causing localized symptoms. Ni, on the other hand, is known to get easily transported via both the xylem and phloem, and to predominantly accumulate in the younger tissues [64]. This behavior explains well the observed accumulation of Ni in the basal parts of daughter fronds, where the cell maturation is still in process.

## 4. Conclusions

This study confirmed that different duckweed species have distinct ionomes, and that ionomic patterns can change considerably due to metal treatments. Duckweed ionomics, therefore, is a valuable approach in exploring factors that affect bioaccumulation of trace pollutants by these plants. Apart from remediating industrial effluents, this aspect may also gain relevance in food and feed safety when duckweed biomass is produced for dietary purposes.

The results also point to the importance of assessing metal accumulating potential on plant area basis. Stress-induced increases in dry matter content and the parallel decrease in horizontal growth can influence the calculated metal uptake efficiency that is traditionally calculated on a dry weight basis. Since the duckweed mat is a rather two-dimensional matrix for wastewater treatment, plant area-based metal accumulation can be at least as relevant as the biomass-based one.

In addition to the mere amount of trace metals removed by duckweed colonies, tracking the internal fate of metallic ions is also important. Our study revealed that within-frond and within-colony compartmentation of metal were strongly metal- and in part species-specific. These results also proved that μXRF can become a useful tool in mapping elemental distributions in duckweed fronds.

## Figures and Tables

**Table 1:** Dry matter content (DM%) of the studied duckweed species on the 3rd day of the applied metal treatments. Means ± SD of *n* = 6 samples; different capitals denote significantly different (*p* < 0.05) median DM% of species with respect to the given treatment (control, Cr(VI) and Ni), while different lower cases indicate significantly different (*p* < 0.05) median DM% of the treatments with respect to the given species.

	Control	Cr(VI)	Ni
*Le. minor*	6.70 ± 0.53 ^Bc^	8.33 ± 0.19 ^Cb^	11.79 ± 0.24 ^Aa^
*La. punctata*	7.26 ± 0.24 ^Bb^	9.11 ± 0.34 ^Ba^	10.02 ± 0.35 ^Ba^
*S. polyrhiza*	8.36 ± 1.01 ^Ac^	11.78 ± 0.96 ^Ab^	13.20 ± 1.09 ^Aa^

## Data Availability

The datasets used in the present study are available from the corresponding author on reasonable request.

## Supplementary Materials

The following is available online at https://www.mdpi.com/article/10.3390/plants12010180/s1, Table S1: ICP-OES settings I., Table S2: ICP-OES settings II., Table S3: Concentration of the analyzed elements in the Steinberg medium on the first and last day of heavy metal treatments, Table S4: Concentrations of the analyzed elements in the biomass of the three tested duckweed species, Table S5: Correlations of the corresponding elements with the principal components.
