# Supplementary material for: Species- and Metal-Specific Responses of the Ionome of Three Duckweed Species under Chromate and Nickel Treatments
Source: Plants (Basel). 2023 Jan 1;12(1):180. doi: 10.3390/plants12010180 (PMC9824728; doi:10.3390/plants12010180)
Supplement: Supplementary file 1 [file plants-12-00180-s001.zip › plants-2082931-supplementary.pdf]

Table S1 ICP-OES settings I.

Replicates	3
Pump speed	15 rpm
Uptake time	15 sec
Rinse time	30 sec
Read time	10 sec
RF power	1.20 kW
Stabilization time	15 sec
Nebulizer flow	0.70 L min^{-1}
Plasma flow	12.0 L min^{-1}
Aux flow	1.0 L min^{-1}
Viewing height	8 mm

Table S2. *Table 2* ICP-OES settings II.

Element	Wavelength	Viewing mode	Stabilization time (sec)
Al	396.152	axial	10
B	249.772	axial	10
Ba	455.403	axial	10
Bi	223.061	axial	10
Ca	445.478	radial	3
Cd	226.502	axial	10
Co	228.615	axial	10
Cr	267.716	axial	10
Cu	324.754	axial	10
Fe	238.204	axial	10
K	766.491	radial	3
Li	670.783	axial	10
Mg	279.800	radial	3
Mn	257.610	axial	10
Na	589.592	radial	3
Ni	216.555	axial	10
Pb	220.353	axial	10
Sr	421.552	axial	10
Zn	213.857	axial	10

Table S3 Concentration (mg l^{-1}) of the analyzed elements in the Steinberg medium on the first (0 day) and last day (3 days) of the heavy metal treatments. Mean±SD of n=6 samples. Bold figures indicate significantly different median concentrations of the respective element within a given treatment (control, Cr(VI) and Ni, respectively) on the 3rd day as compared to the 0th day, according to the Mann-Whitney test. <LoD denotes that the concentration of the given element was below the limit of detection.

		B	Ca	Cr	Cu	Fe	K	Mg	Mn	Na	Ni	Sr	Zn
**control 0 day**		0.084 ±0.002	54.11 ±0.33	<LoD	0.006 ±0.001	0.155 ±0.008	162.6 ±4.7	10.04 ±0.12	0.031 ±0.000	0.635 ±0.014	<LoD	0.014 ±0.000	0.055 ±0.005
**Cr(VI) 0 day**		0.086 ±0.002	53.90 ±0.01	3.668 ±0.046	0.006 ±0.001	0.152 ±0.006	163.0 ±3.6	9.96 ±0.08	0.031 ±0.001	1.034 ±0.152	<LoD	0.014 ±0.000	0.056 ±0.001
**Ni 0 day**		0.084 ±0.003	53.76 ±0.32	<LoD	0.006 ±0.001	0.151 ±0.004	161.2 ±3.5	9.99 ±0.09	0.031 ±0.000	0.635 ±0.008	2.368 ±0.052	0.014 ±0.000	0.069 ±0.008
**Le. minor 3 days**	**control**	0.085 ±0.002	**55.62 ±0.33**	<LoD	0.006 ±0.000	**0.136 ±0.002**	166.3 ±1.4	**10.40 ±0.08**	**0.017 ±0.001**	**0.601 ±0.008**	<LoD	0.014 ±0.001	0.049 ±0.007
	**Cr(VI)**	**0.092 ±0.002**	**55.43 ±0.46**	3.717 ±0.044	0.006 ±0.000	0.147 ±0.001	**169.1 ±1.8**	**10.31 ±0.06**	**0.029 ±0.000**	0.900 ±0.104	<LoD	0.014 ±0.000	0.053 ±0.005
	**Ni**	**0.051 ±0.004**	**55.01 ±0.35**	<LoD	0.005 ±0.001	**0.142 ±0.001**	**166.0 ±1.7**	**10.30 ±0.02**	**0.029 ±0.001**	**0.625 ±0.003**	**2.287 ±0.031**	0.014 ±0.000	**0.044 ±0.003**
**La. punctata 3 days**	**control**	0.086 ±0.004	**55.46 ±0.60**	<LoD	0.006 ±0.001	**0.145 ±0.003**	166.3 ±1.9	**10.35 ±0.10**	**0.014 ±0.002**	0.613 ±0.018	<LoD	0.014 ±0.001	0.051 ±0.005
	**Cr(VI)**	**0.093 ±0.003**	**54.98 ±0.16**	3.704 ±0.047	0.006 ±0.000	0.147 ±0.001	167.5 ±1.8	**10.24 ±0.04**	**0.028 ±0.001**	0.966 ±0.122	<LoD	0.014 ±0.000	**0.051 ±0.002**
	**Ni**	0.085 ±0.002	**55.29 ±0.24**	<LoD	0.006 ±0.001	**0.143 ±0.001**	**166.2 ±1.7**	**10.32 ±0.05**	**0.028 ±0.001**	**0.615 ±0.005**	2.338 ±0.038	0.014 ±0.000	**0.037 ±0.007**
**S. polyrhiza 3 days**	**control**	0.084 ±0.002	**55.24 ±0.39**	<LoD	0.006 ±0.001	**0.133 ±0.002**	164.8 ±2.0	**10.26 ±0.03**	**0.017 ±0.002**	0.627 ±0.009	<LoD	0.014 ±0.000	0.050 ±0.005
	**Cr(VI)**	**0.098 ±0.003**	**55.14 ±0.18**	**3.719 ±0.037**	0.006 ±0.001	0.149 ±0.003	167.6 ±2.0	**10.26 ±0.05**	0.031 ±0.000	1.043 ±0.164	<LoD	0.014 ±0.000	0.062 ±0.006
	**Ni**	0.084 ±0.003	**55.44 ±0.55**	<LoD	0.006 ±0.001	**0.141 ±0.001**	**166.4 ±1.2**	**10.41 ±0.07**	**0.032 ±0.000**	0.639 ±0.014	**2.316 ±0.014**	0.015 ±0.001	**0.057 ±0.004**

Table S4 Concentrations of the analyzed elements in the biomass of the three tested duckweed species on dry weight basis (g kg^{-1} or mg kg^{-1}), on the 3rd day of heavy metal treatments. Mean±SD of n=6 samples. Lower cases denote significantly different ($p<0.05$) median concentrations of the respective element across different treatments (control, Cr(VI) or Ni) with regard to the given duckweed species. Capitals denote significantly different ($p<0.05$) median concentrations of the respective element across different species with regard to the given treatment (control, Cr(VI) or Ni). Figures in red indicate that the concentration of the respective element was below the limit of detection in at least one sample and, therefore, those data were not subjected to statistical comparisons. <LoD indicate that the concentration of the respective element was below the limit of detection in all 6 samples.

Species	treatment	B (mg kg^{-1})	Ca (g kg^{-1})	Cr (mg kg^{-1})	Cu (mg kg^{-1})	Fe (mg kg^{-1})	K (g kg^{-1})	Mg (g kg^{-1})	Mn (mg kg^{-1})	Na (g kg^{-1})	Ni (mg kg^{-1})	Sr (mg kg^{-1})	Zn (mg kg^{-1})	K+Na+Mg+Ca (g kg^{-1})
Le. minor	control	175.3 ±12.7^{bA}	7.22 ±0.67^{cC}	<LoD	6.47 ±1.32^{bAB}	260.9 ±30.5^{aA}	78.9 ±7.5^{aA}	1.71 ±0.14^{bC}	373.3 ±34.1^{aB}	0.74 ±0.06^{bA}	2 ±1	0.826 ±0	253.7 ±45.9^{bA}	88.5 ±8.3^{Aa}
	Cr(VI)	137.4 ±8.8^{cA}	10.79 ±0.28^{aC}	686.6 ±20.2^{A}	2.45 ±0.84^{cB}	109.9 ±14.6b^{AB}	67.6 ±2.2^{bA}	2.00 ±0.04^{aA}	178.9 ±15.7^{bA}	1.87 ±0.19^{aA}	<LoD	0.876 ±0.088	255.7 ±22.3^{bB}	82.2 ±2.4^{Aa}
	Ni	475.9 ±40.9^{aA}	8.50 ±0.68^{bC}	<LoD	14.06 ±1.78^{aA}	135.1 ±35.1^{bB}	59.1 ±3.9^{cA}	2.02 ±0.07^{aA}	147.9 ±20.7^{cB}	0.32 ±0.05^{cB}	1554 ±179^{B}	0.704 ±0.148^{C}	445.5 ±53.1^{aB}	69.9 ±4.5^{Ab}
La. punctata	control	128.4 ±7.1^{bB}	12.08 ±0.68^{aB}	<LoD	5.43 ±0.52^{bB}	128.5 ±23.4^{bB}	57.8 ±2.8^{abB}	1.96 ±0.09^{aB}	456.4 ±39.4^{aA}	0.56 ±0.05^{bB}	6 ±5	1.703 ±0.468^{aB}	308.7 ±22.6^{bA}	72.4 ±3.4^{Ba}
	Cr(VI)	80.1 ±6.4^{cB}	12.87 ±1.23^{aB}	640.3 ±36.3^{B}	3.71 ±2.06^{bAB}	101.7 ±15.0^{cB}	59.5 ±3.3^{aB}	2.03 ±0.12^{aA}	221.9 ±58.7^{bA}	1.11 ±0.19^{aB}	9 ±7	1.595 ±0.481	346.0 ±58.6^{bA}	75.5 ±4.5^{Ba}
	Ni	147.6 ±15.0^{aC}	9.85 ±1.25^{bB}	<LoD	11.84 ±2.26^{aAB}	157.1 ±17.2^{aAB}	49.3 ±6.5^{bB}	1.51 ±0.16^{bB}	184.8 ±19.8^{bA}	0.46 ±0.07^{cA}	1532 ±192^{B}	1.185 ±0.543^{aB}	707.3 ±84.6^{aA}	61.2 ±7.9^{Bb}
S. polyrhiza	control	133.7 ±17.1^{bB}	14.96 ±1.64^{bA}	<LoD	7.25 ±1.21^{bA}	228.0 ±15.9^{aA}	51.5 ±4.8^{abC}	2.29 ±0.20^{aA}	231.1 ±21.5^{aC}	0.25 ±0.07^{bC}	1 ±0	3.247 ±0.860^{aA}	174.7 ±36.0^{bB}	69.0 ±6.4^{Bab}
	Cr(VI)	63.4 ±9.4^{cC}	1.71 ±1.20^{abA}	480.4 ±17.1^{C}	4.19 ±0.86^{cA}	129.8 ±25.4^{bA}	46.9 ±2.9^{bC}	2.13 ±0.19^{aA}	112.4 ±11.1^{bB}	0.34 ±55.6^{aC}	3 ±0	3.303 ±0.537^{a}	144.6 ±30.2^{bC}	66.5 ±4.2^{Cb}
	Ni	263.2 ±13.5^{aB}	18.09 ±1.12^{aA}	<LoD	9.73 ±0.54^{aB}	197.1 ±41.3^{aA}	54.1 ±2.0^{aB}	1.50 ±0.05^{bB}	106.3 ±12.2^{bC}	0.18 ±0.36^{bC}	1840 ±46^{A}	3.257 ±0.647^{aA}	368.7 ±31.6^{aC}	73.9 ±2.9^{Aa}

Table S5 Correlations of the corresponding elements with the principal components

	PC 1	PC 2	PC 3	PC 4	PC 5	PC 6	PC 7	PC 8	PC 9	PC 10	PC 11	PC 12
**B**	0.6962	0.28978	0.11911	0.52976	-0.21835	-0.10033	0.13789	-0.20309	-0.06863	-0.011422	-0.0022718	0.11683
**Ca**	-0.04582	-0.86588	-0.16679	0.10349	0.23275	0.26331	0.18311	-0.058085	-0.024169	0.21978	-0.0025967	0.018053
**Cr**	-0.13652	0.7063	-0.28131	0.25069	0.36024	0.343	-0.15566	-0.24056	-0.077846	-0.030377	-0.028748	-0.056495
**Cu**	0.89258	0.12543	0.2287	0.13733	-0.15772	0.16185	-0.020698	0.14979	-0.12328	0.038467	0.13374	-0.088901
**Fe**	0.093164	-0.044247	0.85293	0.12455	0.37803	-0.036965	-0.29572	0.019651	0.074703	0.07864	0.0015852	0.049897
**K**	-0.36035	0.66903	0.30329	0.39098	0.19414	-0.091247	0.26297	0.22661	-0.0033633	0.040446	-0.079205	-0.057064
**Mg**	-0.48711	-0.25724	-0.12555	0.49753	-0.54707	0.25584	-0.19471	0.15224	0.076605	0.0061193	-0.03668	0.013098
**Mn**	-0.47232	0.43191	0.53174	-0.24855	-0.178	0.37242	0.20322	-0.096992	0.13468	-0.036937	0.066066	0.029751
**Na**	-0.55965	0.53882	-0.5114	0.12534	0.24589	-0.082388	-0.03169	0.14895	0.015454	0.041706	0.13774	0.088489
**Ni**	0.82231	-0.14059	-0.30161	0.21115	0.24367	0.037054	0.057421	0.022843	0.31474	-0.063049	0.010941	-0.027617
**Sr**	-0.23115	-0.81422	0.17018	0.21078	0.33775	0.13809	0.076743	0.10304	-0.11579	-0.21361	0.023475	0.031461
**Zn**	0.7181	0.41206	-0.15616	-0.35224	0.0726	0.29343	-0.0197	0.23501	-0.053053	-0.0025983	-0.088664	0.09171
